# Cerebral hemodynamic effects of early blood pressure lowering after TIA and stroke in patients with carotid stenosis

**DOI:** 10.1177/17474930211068655

**Published:** 2022-01-07

**Authors:** Sara Mazzucco, Linxin Li, Iain J McGurgan, Maria Assuncao Tuna, Nicoletta Brunelli, Lucy E Binney, Peter M Rothwell

**Affiliations:** 1 Wolfson Centre for Prevention of Stroke and Dementia, Nuffield Department of Clinical Neurosciences, University of Oxford, Oxford, UK; 2 Campus Bio-Medico University of Rome, Rome, Italy

**Keywords:** TIA, stroke, blood pressure, transcranial Doppler, carotid stenosis

## Abstract

**Background:**

Effects of early blood pressure (BP) lowering on cerebral perfusion in patients with moderate/severe occlusive carotid disease after transient ischemic attack (TIA) and non-disabling stroke are uncertain.

**Aims:**

We aimed to evaluate the changes in transcranial Doppler (TCD) indices in patients undergoing blood pressure lowering soon after TIA/non-disabling stroke.

**Methods:**

Consecutive eligible patients (1 November 2011 to 30 October 2018) attending a rapid-access clinic with TIA/non-disabling stroke underwent telemetric home blood pressure monitoring (HBPM) for 1 month and middle cerebral artery velocities measurements ipsilateral to carotid stenosis on TCD ultrasound in the acute setting and at 1 month. Hypertensive patients (HBPM ⩾ 135/85) underwent intensive BP-lowering guided by HBPM unless they had bilateral severe occlusive disease (⩾ 70%). Changes in BP and TCD parameters were compared in patients with extracranial moderate/severe carotid stenosis (between 50% and occlusion) versus those with no or mild (< 50%) stenosis.

**Results:**

Of 764 patients with repeated TCD measures, 42 had moderate/severe extracranial carotid stenosis without bilateral severe occlusive disease. HBPM was reduced from baseline to 1 month in hypertensive patients both with versus without moderate/severe carotid stenosis (−12.44/15.99 vs −13.2/12.2 mmHg, respectively, p-difference = 0.82), and changes in TCD velocities (4.69/14.94 vs 2.69/13.86 cm/s, respectively, p-difference = 0.52 for peak systolic velocity and 0.33/7.06 vs 1.75/6.84 cm/s, p-difference = 0.34 for end-diastolic velocity) were also similar, with no evidence of greater hemodynamic compromise in patients with stenosis/occlusion.

**Conclusion:**

There was no evidence of worsening of TCD hemodynamic indices in patients with moderate/severe occlusive carotid disease treated with BP-lowering soon after TIA/non-disabling stroke, suggesting that antihypertensive treatment in this group of patients is safe in the acute setting of TIA clinics.

## Introduction

Blood pressure (BP) lowering after stroke/ transient ischemic attack (TIA) reduces risk of recurrence,^1,2^ but some uncertainty remains around early initiation of antihypertensives in patients with carotid occlusive disease.^3^ There is no benefit, and even possible harm, from early BP-lowering in major acute stroke,^4^ particularly in the subset of patients with moderate/severe carotid stenosis,^5^ but there are few data on effects on cerebral perfusion in TIA and non-disabling stroke patients with carotid stenosis. One study showed no evidence of worsening cerebral perfusion, but numbers were small, patients were enrolled in the post-acute phase and data on perfusion ipsilateral to the arterial stenosis were not provided separately.^3^ In the PROGRESS trial of BP-lowering after stroke/TIA, benefit was seen in patients with large-artery stroke subtype,^6^ but few patients had evidence of carotid stenosis and randomized treatment was delayed until at least 6 weeks after the event.

Uncertainty around timing of antihypertensive treatment initiation and BP targets in TIA/non-disabling stroke patients with carotid stenosis might undermine clinicians’ confidence in early BP-lowering. Failing to prescribe antihypertensive treatment in the acute setting after TIA/non-disabling stroke is a missed opportunity for secondary prevention, as in-hospital prescription is known to be the strongest predictor of long-term adherence.^7–9^ In the randomized trials of endarterectomy in patients with recently symptomatic carotid stenosis, we showed previously that risk of recurrent stroke in the no-surgery group increased with BP level in all but the subgroup with bilateral severe (⩾ 70%) stenosis/occlusion, in which the association was reversed, suggesting that intensive BP-lowering should be avoided in this group, whereas the association remained positive in those with only unilateral ⩾ 70% stenosis.^10^ We, therefore, tested the hypothesis that early BP-lowering would not decrease transcranial Doppler (TCD) blood flow velocities in consecutive patients with recent TIA/non-disabling stroke and ⩾ 50% extracranial carotid stenosis (without bilateral ⩾ 70% stenosis/occlusion) in a population-based cohort attending a rapid-access clinic, and compared results in patients with no significant carotid stenosis.

## Methods

This study was nested in the Oxford Vascular (OxVasc) Study^11^ (Supplementary Methods). OxVasc is approved by the Oxfordshire Research Ethics Committee, and consent is obtained from all participants. From 1 November 2011, all patients attending the OxVasc rapid-access clinic with presumed TIA/non-disabling stroke underwent TCD and telemetric home BP-monitoring (HBPM). Extracranial carotid stenosis (calculated on the basis of the North American Symptomatic Carotid Endarterectomy Trial criteria) was documented through contrast-enhanced magnetic resonance angiography, or if contraindicated, with contrast-enhanced angiographic computed tomography or duplex ultrasound. (Supplementary Methods).

Patients enrolled in the study underwent TCD at two time points: at presentation in the rapid-access TIA/stroke clinic, and at the 1-month follow-up visit. At both time points, TCD sonography (Doppler Box, Compumedics DWL, Singen, Germany) was performed by one of three experienced operators (S.M., M.A.T., and L.L.), who were unaware of the patient’s clinical presentation. Middle cerebral artery (MCA) blood flow velocity was recorded with a handheld 2 MHz probe through temporal bone window at the depth that provided the best signal, usually 50 mm; all parameters were measured twice on each side at both time points and mean values were calculated. TCD examination was conducted in a quiet room, with the patient lying comfortably on a couch, having a lying blood pressure measure taken before and after the scan. Each session was stored in the hard disk of the TCD device for subsequent off-line analysis.

End-tidal CO_2_ was monitored via nasal cannula (Capnocheck Plus; Smith Medical) throughout the procedure at each time point (EtCO_2_—average recorded throughout the TCD procedure).

Patients also underwent three-time daily telemetric BP-monitoring (IEM Stabil-o-Graph or A&D UA-767 BT) each day from baseline assessment to 1-month follow-up (Supplementary Methods). Clinic BP was also assessed at baseline and 1-month follow-up (mean of two readings taken before and after each TCD).^12^

Secondary prevention treatment was started after initial assessment and included aspirin (300 mg loading and then 75 mg daily), plus clopidogrel (300 mg loading dose and then 75 mg for 30 days) for high-risk patients; atorvastatin (40–80 mg daily); antihypertensive treatment (unless systolic blood pressure (SBP) was below 130 mm Hg on repeated measurement), according to a standardized protocol: a combination of perindopril 5 mg and indapamide 1.25 mg followed by addition of amlodipine 5/10 mg, if necessary.^12^ BP-lowering was more cautious in patients with severe bilateral carotid stenosis. These patients were, therefore, excluded from the analysis.

## Statistical analysis

Analysis included all eligible patients recruited until 30 November 2018 who underwent TCD. Hemodynamic TCD measures of peak systolic velocity (PSV), end diastolic velocity (EDV), mean flow velocity (MFV), pulsatility index (PI), and resistance index (RI) were taken ipsilateral to the ⩾ 50% carotid stenosis (either symptomatic or asymptomatic) in patients with carotid stenosis, and compared with the average of parameters from both sides in patients without stenosis. The difference between 1-month follow-up and baseline measure for each variable was expressed as mean (mean 1-month − mean baseline), with negative values reflecting a decrease at 1 month. TCD and BP parameters were compared in patients with (50–100%) versus without (< 50%) carotid stenosis. Analyses were also stratified by hypertensive status based on HBPM on days 0–3 (mean BP ⩾ 135/85 mmHg); sensitivity analyses were done on patients with severe unilateral stenosis (⩾ 70% or occlusion), on patients with recently symptomatic stenosis, and on patients with high pulse pressure (> 60 mmHg). TCD parameters contralateral to carotid stenosis and interhemispheric difference at each time point were also analyzed.

All analyses were done in SPSS version 26 and Stata version 16.1.

## Results

Among 764 patients with repeated TCD measures, reliability of measures at baseline versus 1-month follow-up was good: intra-class correlations (95% CI) 0.88 (0.78–0.94) to 0.91 (0.83–0.95) (Supplementary Table 1).

A total of 697 patients had < 50% carotid stenosis and 67 had ⩾ 50% stenosis/occlusion (Table 1). Of the 67, 13 had ⩾ 70% bilateral ICA stenosis and 13 underwent endarterectomy before 1-month follow-up (Supplementary Tables 2–3), leaving 42 eligible patients with ⩾ 50% stenosis/occlusion for analysis. Of these, 17 had recently symptomatic carotid stenosis (revascularization was not appropriate or declined); 24 of 42 patients displayed unilateral stenosis ⩾ 70%.

**Table 1.** Demographic and clinical characteristics of patients included in the analysis.

	With carotid stenosis		No carotid stenosis (697)
	No CEA^a^ (N = 42)	CEA^a^ (N = 13)	
Age, mean (SD)	75.95 (8.71)	71.08 (9.03)	66.16 (13.91)
Male sex	26 (62)	7 (54)	414 (53.1)
Time between symptoms onset and first assessment (median days, IQR)	3 (2–11.25)	3 (2–5.5)	3 (2–9)
Diagnosis			
TIA	25 (60)	9 (69)	433 (62.1)
Stroke	16 (38)	4 (31)	212 (30.4)
Mimics	1 (2)	0	52 (7.5)
Risk factors			
History of hypertension	29 (78)	11 (85)	330 (47.3)
History of smoking	25 (66)	9 (69)	354 (50.8)
History of hyperlipidemia	17 (46)	8 (67)	218 (31.3)
History of atrial fibrillation	4 (11)	1 (8)	71 (10.2)
History of diabetes	7 (19)	2 (15)	75 (10.8)
DWI-positive on acute MRI imaging^b^	8 (30)^c^	7 (58)	103 (18.2)
Median mRS follow-up (IQR)	1 (1,2)	1 (1,2)	1 (0,2)

CEA: carotid endarterectomy; SD: standard deviation; IQR: interquartile range; TIA: transient ischemic attack; DWI: diffusion-weighted imaging; MRI: magnetic resonance imaging; mRS: modified Rankin Scale.

Variables are expressed as N (%) unless stated otherwise.

a CEA/stent between baseline and follow-up.

b Patients with DWI-positive lesion/patients who had MRI imaging in the acute phase.

c DWI lesions were ipsilateral to the carotid stenosis apart from two cases with asymptomatic stenosis.

A total of 34 (81%) patients were already on some BP-lowering medication at baseline. Of the 25 patients who remained hypertensive at baseline, 17 (80%) agreed to medication being initiated or increased.

**Figure 1.**
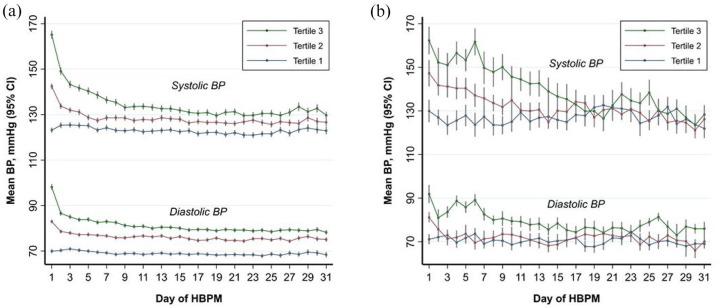
Systolic and diastolic BP on HBPM in (a) the group of patients with mild/no carotid stenosis and (b) in patient with ⩾ 50% carotid stenosis/occlusion, during the first month after clinical assessment, by tertiles.

**Figure 2.**
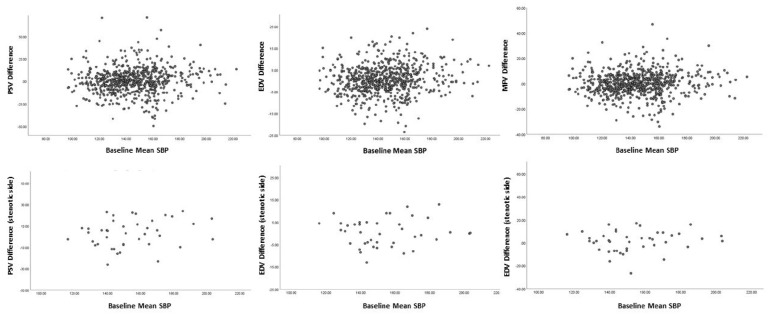
Correlation between baseline SBP and mean TCD velocities changes between baseline and follow-up in patients with carotid stenosis (second line) who did not undergo carotid endarterectomy between baseline and follow-up (excluded patients with severe bilateral stenosis) versus those with mild/no carotid stenosis (first line).

A similar decrease in SBP on HBPM between baseline and follow-up (Figure 1, Tables 2 and 3) was observed both in patients with and without carotid stenosis, particularly in those who were hypertensive on HBPM during days 0–3 (−12.44/15.99 vs −13.2/12.2 mmHg, respectively, p-difference = 0.82; Table 3). TCD parameters did not change significantly between baseline and follow-up in patients with ⩾ 50% carotid stenosis (Table 2 and Supplementary Table 4), and were similar to those with no/< 50% stenosis. (Table 3, Figure 2).

**Table 2.** Physiological variables and hemodynamic parameters in patients with ipsilateral moderate/severe carotid artery stenosis at baseline, follow-up, and difference between the two time points.

	Physiological variable	Baseline mean/SD	1 Month FU mean/SD	Difference mean/SD	p
Carotid stenosis without CEA (42)	SBP (mmHg)	154.05/20.70	147.14/20.76	−6.90/18.10	0.018
	DBP (mmHg)	81.10/12.94	73.83/8.58	−7.26/11.54	< 0.0001
	PP (mmHg)	72.95/17.41	73.31/17.53	0.36/13.28	0.862
	EtCO_2_ KPa	4.82/0.81	5.02/0.93	0.19/0.66	0.161
	PSV (cm/s)	83.40/21.66	86.57/22.40	3.17/12.91	0.124
	EDV (cm/s)	29.93/9.64	30.41/9.79	0.48/6.08	0.615
	MFV (cm/s)	49.98/13.71	51.35/13.80	1.37/9.15	0.335
	PI	1.11/0.24	1.10/0.24	−0.005/0.15	0.816
	RI	0.64/0.08	0.64/0.08	−0.003/0.05	0.638

FU: follow up; CEA: carotid endarterectomy; SBP: clinic systolic blood pressure; DBP: clinic diastolic blood pressure; PP: pulse pressure; EtCO_2_: end-tidal CO_2_; PSV: peak systolic velocity; EDV: end-diastolic velocity; MFV: mean flow velocity; PI: pulsatility index; RI: resistance index.

**Table 3.** SBP and TCD parameters changes during the first month after TIA/stroke by HBPM hypertension classification in patients with moderate/severe carotid stenosis (excluding patients with bilateral severe carotid stenosis) who did not undergo carotid endarterectomy (CEA) versus patients with mild/no carotid stenosis.

Table 1	N	Baseline to 1-month change mean /SD	p	N	Baseline to 1-month change mean (SD)	p	p-difference
Carotid stenosis (No CEA)				No carotid stenosis—whole cohort			
SBP (mmHg)	42	−6.90/18.10	0.018	697	−11.3/19.9	< 0.001	0.13
PSV (cm/s)		3.08/12.92	0.135		0.88/13.8	0.096	0.29
EDV (cm/s)		0.44/6.13	0.649		0.77/7.26	0.005	0.73
MFV (cm/s)		1.32/9.15	0.357		0.32/9.41	0.375	0.49
PI		−0.005/0.15	0.816		−0.004/0.14	0.476	0.97
RI		−0.006/0.05	0.638		−0.005/0.05	0.016	0.90
Carotid stenosis with hypertension (HBPM ⩾ 135/85) (No CEA)				No carotid stenosis—hypertension (HBPM ⩾ 135/85)			
SBP (mmHg)	25	−12.44/15.99	0.001	226	−13.2/12.2	< 0.001	0.82
PSV (cm/s)		4.69/14.94	0.138		2.68/13.86	0.004	0.52
EDV (cm/s)		0.33/7.06	0.819		1.75/6.84	< 0.001	0.34
MFV (cm/s)		1.48/10.81	0.500		1.34/8.96	0.025	0.95
PI		0.03/0.12	0.193		−0.01/0.16	0.273	0.13
RI		0.003/0.04	0.775		−0.008/0.06	0.049	0.22

SD: standard deviation; CEA: carotid endarterectomy; SBP: clinic systolic blood pressure; PSV: peak systolic velocity; EDV: end-diastolic velocity; MFV: mean flow velocity; PI: pulsatility index; RI: resistance index; HBPM: home blood pressure monitoring.

The sensitivity analysis on patient with more severe stenosis and in those with symptomatic carotid stenosis showed that BP reduction in these groups was overall more cautious. However, changes in TCD parameters were qualitatively similar in patients with moderate (50–69%) and severe stenosis (⩾ 70/occlusion) and in those with symptomatic stenosis, including in the subgroups where BP was significantly reduced between baseline and follow-up (Supplementary Tables 5–7).

Further sensitivity analyses are shown in Supplementary Tables 8–9.

There were no early recurrences (within 1 month from the event) in the 42 patients with carotid stenosis.

## Discussion

Our study showed no detrimental effects of early BP-lowering soon after TIA/non-disabling stroke on TCD velocities in patients with moderate/severe carotid occlusive disease when compared with patients with no or mild (< 50%) stenosis. This finding is in line with the risk associations reported in previous endarterectomy trials on recently symptomatic carotid stenosis.^10^

Taken together, these observations should encourage clinicians to treat hypertension after TIA/non-disabling stroke in patients with moderate/severe carotid stenosis/occlusion, unless they have bilateral severe occlusive disease (⩾ 70% or occlusion).

Our study has several strengths. It is the first such study in consecutive unselected patients treated early after TIA/non-disabling stroke; effective BP-lowering was documented using HBPM; and the follow-up TCD measures were done after 1 month, when any cerebral dysregulation is likely to have resolved, with parameters analyzed ipsilaterally to the stenotic side. However, our study has some weaknesses. First, we used TCD sonography, which provides a measure of blood flow velocity in the basal cerebral arteries rather than of blood flow.^13^ Furthermore, TCD parameters measured on the proximal MCA do not necessarily reflect more regional effects of BP lowering throughout the brain. Patients with carotid stenosis might have coexisting posterior circulation stenosis; there might be a difference between anterior and posterior circulation response to BP lowering in these patients that might not be captured in the MCA. Moreover, collateral perfusion or coexisting extensive small vessel disease might contribute to regional differences in response to blood pressure lowering. However, other imaging methods, including Xe inhalation method,^14^ single photon emission computed tomography,^15^ arterial spin labeling magnetic resonance imaging,^16^ are less practical in large studies and more likely to lead to exclusion of clinically complex and frail patients. TCD is an accurate and easily accessible method for functional studies of cerebral hemodynamics, and TCD flow velocities are strongly correlated with blood flow volume in the proximal MCA,^17–19^ with negligible changes in diameter in the proximal MCA secondary to pharmacologically induced BP reductions.^19,20^ Second, although group-mean TCD velocities tended to increase between baseline and follow-up, there was variation between patients. However, the range of changes in the group of patients with carotid stenosis was similar to that of patients without carotid stenosis, with no patient exceeding the limits of variation in this group. Finally, BP reduction was more cautious in patients with unilateral severe (⩾ 70%) carotid stenosis/occlusion and in those with recently symptomatic stenosis. However, TCD changes in these groups were similar to those observed in patients with moderate (50%) stenosis, even in the subgroup of patients with severe/symptomatic stenosis undergoing intensive BP reduction between baseline and follow-up.

## Conclusion

We showed no evidence of worsening of TCD hemodynamic parameters in patients with ⩾ 50% carotid stenosis treated with BP-lowering soon after TIA/non-disabling stroke, suggesting that antihypertensive treatment in this group is safe in the acute setting. Further data are needed for patients with severe bilateral carotid stenosis.

## Supplemental Material

sj-docx-1-wso-10.1177_17474930211068655 – Supplemental material for Cerebral hemodynamic effects of early blood pressure lowering after TIA and stroke in patients with carotid stenosis Click here for additional data file. Supplemental material, sj-docx-1-wso-10.1177_17474930211068655 for Cerebral hemodynamic effects of early blood pressure lowering after TIA and stroke in patients with carotid stenosis by Sara Mazzucco, Linxin Li, Iain J McGurgan, Maria Assuncao Tuna, Nicoletta Brunelli, Lucy E Binney and Peter M Rothwell in International Journal of Stroke
